# Association between perception of care coordination and health outcomes in Korean cancer survivors

**DOI:** 10.1186/s12955-020-1279-6

**Published:** 2020-02-04

**Authors:** Jinyoung Shin, Dong Wook Shin, Jungkwon Lee, Ji Hye Hwang, Jung Eun Yoo, Hyeonyoung Ko, Yun-Mi Song

**Affiliations:** 1 Department of Family Medicine, Konkuk University Medical Center, Konkuk University School of Medicine, 120-1, Neungdong-ro, Gwangjin-gu, Seoul, 05030 South Korea; 2 Department of Family Medicine, Samsung Medical Center, Sungkyunkwan University School of Medicine, 81 Irwon-ro, Gangnam-gu, Seoul, 06351 South Korea; 3 Department of Physical and Rehabilitation Medicine, Samsung Medical Center, Sungkyunkwan University School of Medicine, 81 Irwon-ro, Su Gangnam-gu, Seoul, 06351 South Korea; 4 Department of Family Medicine, Healthcare System Gangnam Center, Seoul National University Hospital, 152, Teheran-ro, Gangnam-gu, Seoul, 06236 South Korea; 5 Department of Family Medicine, Kangbuk Samsung Hospital, Sungkyunkwan University School of Medicine, 29 Saemunan-ro, Jongno-gu, Seoul, 03181 South Korea

**Keywords:** Disease management, Cancer survivors, Comorbidity, Quality of life, Patient satisfaction

## Abstract

**Background:**

To determine relationship between cancer survivors’ perception of care coordination and their health outcomes.

**Methods:**

Study subjects were 1306 Korean adulthood cancer survivors who were enrolled in two academic hospital and completed a questionnaire consisting of questions asking two aspects of care coordination for cancer treatment they had received: 1) who played a main coordinator role and 2) whether care services had met their necessitated health concerns. We measured health outcomes including new comorbidity, number of clinic visits, health-related quality of life (HRQoL) and fear of cancer recurrence (FCR). Associations between the level of care coordination and health outcomes were evaluated by multiple logistic regression analysis after adjusting for covariates.

**Results:**

Survivors with uncoordinated care were more likely to have more new comorbidities after cancer diagnosis, visit clinic more frequently and have worse HRQoL and higher FCR. Females and unmarried survivors were more likely to have received uncoordinated care than males and ever married survivors. Uncoordinated care group had an increased the risk of new comorbidity (odds ratio 1.73, [95% confidence interval] 1.02–2.92), multiple clinic visits (1.69, 1.00–2.88), severe FCR (2.28, 1.33–3.93), low EuroQoL Visual Analogue Scale (1.82, 1.28–2.60), low global health status (1.51, 1.04–2.21), and poor physical (2.00, 1.31–3.04), role (2.46, 1.69–3.56) and emotional function (2.62, 1.81–3.78).

**Conclusions:**

Coordinated care of Korean cancer survivors was associated with their health outcomes, including new comorbidity, clinic visits, HRQoL and FCR. Good care coordination may be reinforced to improve outcomes of survivorship care.

## Background

The number of Korean cancer survivors is dramatically increasing. It was estimated to be 1.6 million in 2015 [1]. Post-treatment period of cancer patients is characterized by gradual recovery from many treatment-related adverse effects. However, many cancer survivors still suffer from various health issues including long-term and late effects of treatment through their survivorship journey [2]. The care of cancer survivors is very likely to be provided by healthcare professionals of several different health disciplines, making survivorship care very complicated [3]. Therefore, care coordination is a very important issue in cancer survivorship care [4].

The Agency for Healthcare Research and Quality defines care coordination as follows: “Care coordination is the deliberate organization of patient care activities between two or more participants (including the patient) involved in a patient’s care to facilitate the appropriate delivery of health care services” [3, 5]. A systematic review of 52 studies including 598,683 participants on effects of cancer care coordination has found that care coordination could lead to improvement of appropriate health care utilization and care cost reduction [3]. Another systematic review has found that most coordinating actions that influence health outcomes of cancer patients are focused on information sharing or monitoring within a given care team or group [6].

However, the effectiveness of care coordination has been evaluated mainly about healthcare professionals-related outcomes such as medication error, avoidable hospitalization, or emergency department visiting [3, 6–10]. There is a lack of evidence about processes or integrating conditions that can mediate the relationship between care coordination and patient outcomes [3, 11]. Moreover, most previous studies were from Western countries [3, 6, 12, 13]. Thus, it is difficult to apply processes and structures of care coordination found in those studies to countries with different medical setting and culture.

In Korea, instead of multidisciplinary team-based cancer care, surgical and medical oncologists are usually responsible for the whole cancer treatment process, including surveillance and supportive care even after completing cancer treatment. Therefore, most Korean cancer patients try to discuss their health issues with oncologists. However, the oncologists hardly provide patients with high-quality supportive care. They tend to just refer patients to multiple other specialists within the same hospital to deal with patients’ request due to insufficient consultation time and excessive workload. This may lead cancer patients to receive fragmented care [14]. In addition, Korean patients have freedom of choice in selecting healthcare providers and institutions because the healthcare delivery system based on primary care has not been well-established in Korea [15]. Thus, lots of Korean cancer patients whose needs are not met may visit several specialists without proper professional guidance [15, 16]. Along with increasing importance of care transition to primary care setting these days [16], effects of care coordination on clinical outcomes, quality of life and health care utilization need to be evaluated in Korea cancer patients. Thus, the objective of this study was to evaluate the association of perception of care coordination that they experienced through their cancer journey and their health outcomes including comorbidity, health-related quality of life (HRQoL), use of medical care and fear of cancer recurrence by enrolling Korean adulthood cancer survivors.

## Methods

### Study participants

We recruited a total of 1486 adult (≥ 19 years) cancer survivors who visited hospital-based cancer survivorship clinics in two university affiliated hospitals from September 2014 to March 2017. The two institutions run separate cancer center and have been involved in cancer care of around 20% of Korean cancer patients. Most of them visited the clinic for the first time due to their unmet health concerns or for post-treatment surveillance after 5 years from their initial cancer diagnosis. Among them, 180 subjects were excluded because of the following reasons: within one-year of cancer diagnosis time (*n* = 105), lack of information for study variables (*n* = 62), or metastasis at diagnosis (*n* = 13). Thus, 1306 survivors were finally included in the present study.

### Study variables

Demographic and socioeconomic characteristics were surveyed using a self-administered questionnaire and categorized as follows: monthly income level (≥ 4,000,000 won, 2,000,000-3,999,999, < 2,000,000 and unknown), education level (≤ 9 years, 10–12 and ≥ 13) and marital status (married/with partner, unmarried and divorce/bereavement). A trained research assistant supplemented incompletely answered questions through additional face-to-face interview if necessary. We reviewed medical records to obtain information about cancer, including site, time since cancer diagnosis (< 1 year, 1–5 year, or > 6 years), stage (I, II, III, or IV), treatment modality, status of metastases at the time of primary cancer diagnosis and cancer recurrence.

We collected information on comorbidity diagnosed by physicians, including the time of comorbidity development. Any chronic disease diagnosed by physician at the first time after cancer diagnosis was categorized as ‘new comorbidity after cancer diagnosis’, including various diseases such as cerebrovascular disease, hypertension, diabetes mellitus, dyslipidemia, mental health disorder (depression, anxiety) and others; thyroid disease, liver disease, lung disease, osteoporosis, lymphedema, peripheral neuropathy and anemia. We also surveyed the number of clinic visits per year regardless of the clinical cause (cancer or non-cancer).

We assessed the perception of care coordination for cancer treatment that subjects had received previously using the following two questions chosen from the ‘Site self- assessment evaluation tool for the Maine health access foundation integration initiative’ [17]. The first question was: “Who played a care coordinator role for your cancer treatment? By yourself or medical team?” It had five levels of responses ranging from 1 (always by medical care team) to 5 (always by myself with lots of difficulties). The second question was: “Did you receive all care services that were necessary for dealing with your health concern?” It also had five levels of responses, ranging from 1 (absolutely no) to 5 (absolutely yes). Using responses to these two questions, we categorized study subjects into three groups of perception to care coordination (coordinated, intermediate, uncoordinated). Coordinated group included subjects who responded that ‘care plan was mostly provided by medical team’ and that ‘I received care services had met my necessitated health concerns’. ‘Uncoordinated’ group included subjects who responded that ‘care plan was mostly provided by myself’ and that ‘I never received needed care services’. The remaining subjects were categorized into ‘intermediate’ group. Study subjects who did not need any supportive care (*n* = 44) were classified into ‘coordinated’ group. We assessed study subjects’ perception about the level of whole person care including physical, psychological, social and spiritual care provided by the medical team and the levels of communication provided by the medical team. We assessed study subjects’ perception about the level of communication provided by the medical team by summation of their responses to three relevant questions (harmony, interaction and role). We also assessed whether study subjects were provided with tailored education for life style modification such as healthy diet, weight control, physical activity, alcohol intake and smoking in their cancer care journey [17]. Type of coordinated care providers other than oncologists were also assessed such as physicians in supportive center or family physicians, physicians of other specialty, dietitian or counsellor, or nurse practitioners.

The EuroQoL-visual analogue scale (EQ-VAS) and the European Organization for Research and Treatment of Cancer Quality of Life Questionnaire version 3.0 (EORTC QLQ-C30) were used to assess HRQoL. The EQ-VAS is a standard vertical 20-cm visual analogue scale for rating current HRQoL, ranging from zero (worst imaginable) to 100 (best imaginable). EORTC QLQ-C30 is a 30-item questionnaire developed to assess health related quality of life of cancer patients, incorporating five functional scales (physical, cognitive, social, emotional and role), symptom scale and global quality of life scale. The score of each scale ranged from zero to 100. Cronbach’s alpha coefficients for the Korean version of EORTC QLQ-C30 have been found to be greater than 0.70 for most subscales in a previous validation study except for cognitive functioning (α = 0.60) [18].

Fear of Cancer Recurrence Index (FCRI) is a multidimensional questionnaire composed of 42 items with seven subscale components of fear of cancer recurrence (FCR) [19]. Its components were: potential stimuli activating FCR (triggers), presence and severity of intrusive thoughts associated with FCR (severity), emotional disturbance associated with FCR (psychological distress), impact of FCR on important areas of functioning (functional impairments), self-criticism toward FCR intensity (insight), reassurance seeking such as thorough self-examination or repeated medical consultations (reassurance) and other strategies to cope with FCR (coping strategies). FCRI ranged from zero to 168. Cronbach’s alpha coefficient for the K-FCRI was 0.85 for total scale and 0.77–0.87 for subscales [19].

### Statistical analysis

Descriptive statistics were presented as a summary of clinical and socioeconomic characteristics of study participants according to three levels of cancer care coordination with comparisons among three groups using one-way analysis of variance (ANOVA) for continuous variables and Chi-square test for categorical variables.

We evaluated relations of care coordination for content-specific agreement, clinical and psychosocial outcomes, and quality of life. *P* values for trend were obtained using linear regression analysis for continuous variables and Mantel-Haenzel Chi-square test for categorical variables.

Multiple logistic regression analysis was performed to assess how with the level of care coordination was associated with comorbidity development and medical use after adjusting for age, sex, cancer site, marital status, cancer stage, treatment modality, type of care providers and duration since cancer diagnosis. As we arbitrarily set cut-off point of outcome variables based on their quartile distribution, we did sensitivity analysis using linear regression analysis from which β coefficients (95% confidence interval) for log-transformed HRQoL and FCRI associated with increased level of coordinated care were estimated. Then, percent difference of HRQoL and FCRI was calculated by multiplying 100 to the value of exponentiated β coefficient − 1.

All analyses were performed using PASW Statistics 23.0 software (SPSS Inc., Chicago, IL, USA).

## Results

Among 1306 study subjects, 49.4% were categorized as ‘coordinated group’ and 12.8% were categorized as ‘uncoordinated group’ based on the care coordination. The mean age of study subjects was 57.3 years (range, 23–91 years). Among total participants, 62.2% were females. Stomach and breast were the most common cancer sites. The most common cancer stage at the time of diagnosis was stage I (81.5%). When we compared some demographic and clinical characteristics of participants (*n* = 1306) and non-participants who refused to participate in our study (*n* = 268), there was no significant difference in age, sex, time lapse since cancer diagnosis, and treatment modality (Additional file 1: Table S1).

Distributions of levels of satisfaction with care coordination according to clinical and socio-demographic characteristics are shown in Table 1. The level of care coordination was found to vary according to age (at survey and cancer diagnosis), sex, cancer sites, treatment modality, duration since cancer diagnosis, marital status and type of care providers other than oncologists. Cancer survivors who were younger, females, diagnosed with cancer within the last 5 years and without a marital partner were more likely to be classified into the uncoordinated group. However, the distribution of level of care coordination did not differ according to cancer stage, house income, or achieved education level.

**Table 1:** Clinical and socio-demographic characteristics of study subject according to the level of care coordination

Level of care coordination					
Variables	Overall (*N* = 1306)	Uncoordinated group (*N* = 167)	Intermediate group (*N* = 494)	Coordinated group (*N* = 645)	*P*-value*
Age at survey	57.3 ± 12.5	56.1 ± 9.1	55.2 ± 15.0	59.3 ± 10.6	< 0.001
Age at cancer diagnosis	51.9 ± 12.2	50.8 ± 8.6	49.9 ± 15.2	53.8 ± 9.9	< 0.001
Sex					< 0.001
Male	494	50 (10.1)	162 (32.8)	282 (57.1)	
Female	812	117 (14.4)	332 (40.9)	363 (44.7)	
Cancer site					< 0.001
Stomach	527	47 (8.9)	166 (31.5)	314 (59.6)	
Breast	360	61 (16.9)	151 (41.9)	148 (41.1)	
Lung	119	14 (11.8)	44 (37.0)	61 (51.3)	
Thyroid	93	18 (19.4)	39 (41.9)	36 (38.7)	
Colon/rectum	56	8 (14.3)	28 (50.0)	20 (35.7)	
Prostate	33	4 (12.1)	9 (27.3)	20 (60.6)	
Others	118	15 (12.7)	57 (48.3)	46 (39.0)	
Cancer stage					0.570
I	1064	137 (12.9)	394 (37.0)	533 (50.1)	
II	148	21 (14.2)	60 (40.5)	67 (45.3)	
III, IV	94	9 (9.6)	40 (42.6)	45 (47.9)	
Treatment modality					0.012
Surgery	447	63 (14.1)	193 (43.2)	191 (42.7)	
Surgery+CT	105	11 (10.5)	47 (44.8)	47 (44.8)	
Surgery+CT + RT	97	9 (9.3)	40 (41.2)	48 (49.5)	
Surgery+CT + RT + HT	48	7 (14.6)	22 (45.8)	19 (39.6)	
Others	609	77 (12.6)	192 (31.5)	340 (55.8)	
Duration since cancer diagnosis					0.004
1–5 year	377	57 (15.1)	146 (38.7)	174 (46.2)	
> 6 year	929	110 (11.8)	348 (37.5)	471 (50.7)	
House income (won/month)					0.110
≥ 4,000,000	454	66 (14.5)	191 (42.1)	197 (43.4)	
2,000,000-3,999,999	263	29 (11.0)	120 (45.6)	114 (43.3)	
< 2,000,000	241	44 (18.3)	87 (36.1)	110 (45.6)	
Unknown	348	28 (8.0)	96 (27.6)	224 (64.4)	
Achieved education level					0.301
0–9 years	579	78 (13.5)	233 (40.2)	268 (46.3)	
10–12 years	541	86 (15.9)	233 (43.0)	222 (41.1)	
≥ 13 years	186	19 (10.1)	75 (40.4)	92 (49.5)	
Marital status					0.014
Married/with partner	1129	130 (11.5)	426 (37.7)	573 (50.8)	
Divorce/bereavement	113	20 (17.7)	40 (35.4)	53 (46.9)	
Unmarried	64	17 (26.6)	28 (43.8)	19 (30.0)	
Type of care providers other than oncologists					< 0.001
Physicians in supportive center or family physician	165	22 (8.3)	69 (41.8)	74 (44.8)	
Physicians of other specialty	54	8 (14.8)	8 (14.8)	38 (70.4)	
Dietitian/Counsellor	159	20 (12.6)	67 (42.1)	72 (45.3)	
Nurse practitioner	57	7 (12.3)	21 (36.8)	29 (50.9)	

Data were presented as mean value ± standard deviation or number (row percentage)

*CT* chemotherapy, *RT* Radiotherapy, *HT* hormone therapy

*P values were obtained by one-way ANOVA analysis of variance test or chi-square test

Table 2 shows how care coordination is associated with the perception of whole person care, communication with the medical team and receipt of tailored patient education for lifestyle modification. The level of care content-specific agreement tended to increase with increasing level of care coordination across all specific areas of care (*p* < 0.05). The uncoordinated group was less likely to have received whole person care across all areas compared to the coordinated group. The uncoordinated group was less likely to agree to good communication with the medical team compared to the coordinated group. Overall, the most common content of tailored patient education was healthy-diet (64.1%), followed by weight control (58.4%) and physical activity (58.1%). Alcohol intake (27.4%) and smoking (26.1%) were less commonly dealt with as contents of patient education relatively. The coordinated group was most likely to receive patient education across all lifestyle modification areas.

**Table 2:** Care content-specific agreement of study subjects according to the level of care coordination

Care contents	Level of care coordination				P-for trend†
	Overall (*N* = 1306)	Uncoordinated group (*N* = 167)	Intermediate group (*N* = 494)	Coordinated group (*N* = 645)	
Whole person care (1–5)^a^					
Physical care	3.6 ± 1.1	3.0 ± 1.1	3.3 ± 1.0	4.0 ± 1.0	< 0.001
Psychological care	3.2 ± 1.2	2.7 ± 1.2	2.9 ± 1.0	3.6 ± 1.2	< 0.001
Social care	2.3 ± 1.3	1.8 ± 1.2	2.0 ± 1.0	2.6 ± 1.4	< 0.001
Spiritual care	2.2 ± 1.3	1.8 ± 1.2	1.9 ± 1.0	2.4 ± 1.4	< 0.001
Communication within medical team (3–15)^a^	14.2 ± 1.6	13.4 ± 2.3	14.1 ± 1.4	14.4 ± 1.5	< 0.001
Tailored patient education contents, yes					
Diet	64.1	52.1	59.6	70.7	< 0.001
Weight control	58.4	45.5	55.7	63.8	< 0.001
Physical activity	58.1	48.5	55.7	62.5	0.021
Alcohol intake	27.4	20.5	22.6	33.1	0.014
Smoking	26.1	20.5	20.7	31.7	0.017

Data were presented as mean value ± standard deviation or percentage

^a^ Range of score. Higher score means higher level of agreement

† *P* values for trend were obtained by linear regression analysis for continuous variables and Mantel-Haenzel chi-square test for categorical variables

Compared to the distribution of clinical and psychosocial outcomes, HRQoL and K-FCRI according to the level of care coordination are presented in Table 3. We found that the prevalence of comorbidity has increased by 12.5% since the time of cancer diagnosis, and the increase was especially marked in uncoordinated care group (17.9%) compared to intermediate group (11.3%) or coordinated group (12.1%) (Additional file 1: Table S2). In Table 3, the number of comorbidities in a survivor ranged from 0 to 4, with a mean number of new comorbidities of 0.42 (30.3% of participants). Although the prevalence of comorbidity prior to cancer diagnosis did not differ by the level of care coordination (data not shown), the uncoordinated group was more likely to develop a new comorbidity after cancer diagnosis than the other two groups (*p* for trend: 0.039). The mean number of clinic visits was 2.4 times per year and the maximum number of clinic visits was 14 (one participant). The uncoordinated group visited clinics more frequently than the coordinated group. The uncoordinated group showed worse HRQoL measured by EQ-VAS and global, functional and symptom scales of EORTC QLQ-C30. The uncoordinated group also showed higher level of trigger, severity, insight and psychological distress of FCR than the coordinated group. However, financial difficulty in EORTC QLQ-C30, functional impairment, or reassurance in K-FCRI was not significantly associated with the level of care coordination.

**Table 3:** Clinical and psychosocial outcomes and quality of life according to the level of care coordination

Measurement tools	(range)	Overall (*N* = 1306)	Uncoordinated group (*N* = 167)	Intermediate group (*N* = 494)	Coordinated group (*N* = 645)	*P*-for trend*
		N (%) Mean ± SD	N (%) Mean ± SD	N (%) Mean ± SD	N (%) Mean ± SD	
New comorbidity after cancer diagnosis		396 (30.3)	55 (32.9)	159 (32.2)	182 (28.2)	0.039
Type of new Comorbidity						< 0.001
Cerebrovascular		16 (1.2)	3 (1.8)	8 (1.6)	5 (0.8)	
Hypertension		65 (5.0)	10 (6.0)	26 (5.3)	29 (4.5)	
Diabetes mellitus		23 (1.8)	1 (0.6)	9 (1.8)	13 (2.0)	
Dyslipidemia		105 (8.0)	15 (9.0)	39 (7.9)	51 (7.9)	
Mental disorder		14 (1.1)	2 (1.2)	4 (0.8)	8 (1.2)	
Others^a^		173 (13.2)	24 (14.4)	73 (14.8)	76 (11.8)	
No of new comorbidity	0–4	0.42 ± 0.72	0.44 ± 0.75	0.48 ± 0.79	0.37 ± 0.66	0.012
No of clinic visiting, yearly	1–14	2.4 ± 1.6	2.8 ± 1.9	2.5 ± 1.5	2.3 ± 1.6	0.004
EQ_VAS^b^	0–100	68.1 ± 16.7	64.6 ± 18.1	67.1 ± 17.3	69.8 ± 15.7	< 0.001
EORTC QLQ-C30						
*Global health status/QOL*	0–100	66.4 ± 18.7	63.7 ± 20.0	64.8 ± 19.0	68.4 ± 17.9	0.001
*Functional scales*^b^						
Physical functioning	0–100	81.1 ± 16.2	77.4 ± 15.7	80.1 ± 16.4	82.8 ± 16.0	< 0.001
Role functioning	0–100	86.4 ± 20.1	81.0 ± 22.5	83.5 ± 22.0	90.0 ± 17.0	< 0.001
Emotional functioning	0–100	80.0 ± 20.2	73.1 ± 22.5	76.1 ± 21.1	84.8 ± 17.6	< 0.001
Cognitive functioning	0–100	75.6 ± 19.3	71.4 ± 19.2	74.8 ± 19.7	77.3 ± 18.9	0.001
Social functioning	0–100	80.0 ± 23.2	77.3 ± 25.6	76.8 ± 23.7	83.1 ± 21.7	< 0.001
*Symptom scales*^c^						
Fatigue	0–100	33.8 ± 24.6	43.1 ± 24.1	37.4 ± 25.9	28.7 ± 22.4	< 0.001
Nausea and vomiting	0–100	10.3 ± 16.3	14.4 ± 19.9	11.7 ± 17.2	8.1 ± 14.2	< 0.001
Pain	0–100	17.8 ± 22.0	21.6 ± 22.9	21.4 ± 24.1	14.1 ± 19.2	< 0.001
Dyspnea	0–100	16.7 ± 22.3	23.6 ± 24.9	19.6 ± 22.4	12.7 ± 20.7	< 0.001
Insomnia	0–100	29.5 ± 32.1	38.5 ± 31.9	30.2 ± 31.3	26.6 ± 32.3	< 0.001
Appetite loss	0–100	13.9 ± 24.0	20.8 ± 24.4	14.9 ± 27.5	11.4 ± 20.3	< 0.001
Constipation	0–100	18.6 ± 25.1	25.0 ± 26.8	21.7 ± 26.3	14.6 ± 23.1	< 0.001
Diarrhea	0–100	21.7 ± 25.3	26.1 ± 29.1	23.5 ± 25.5	19.2 ± 23.8	< 0.001
Financial difficulties	0–100	18.4 ± 26.9	20.6 ± 29.9	19.7 ± 27.0	16.9 ± 25.9	0.086
K-FCRI						
Triggers^c^	0–32	13.1 ± 6.8	15.1 ± 7.3	13.6 ± 7.0	12.0 ± 6.3	< 0.001
Severity^c^	0–36	11.2 ± 7.3	13.6 ± 7.4	12.4 ± 7.2	9.6 ± 6.9	< 0.001
Psychological distress^c^	0–16	4.2 ± 3.9	5.3 ± 3.9	4.6 ± 4.1	3.5 ± 3.6	< 0.001
Functional impairment^c^	0–24	5.0 ± 5.4	5.2 ± 5.2	5.1 ± 5.7	4.9 ± 5.3	0.920
Coping strategies^b^	0–36	19.1 ± 7.6	18.3 ± 8.2	18.6 ± 7.1	19.9 ± 7.6	0.005
Insight^c^	0–12	1.3 ± 2.1	1.8 ± 2.7	1.6 ± 2.2	1.0 ± 1.8	0.014
Reassurance^b^	0–12	5.0 ± 3.2	4.7 ± 2.9	4.9 ± 3.2	5.1 ± 3.4	0.129

Data were presented as mean value ± standard deviation, number, or number (percentage)

*EQ-VAS* EuroQoL Visual Analogue Scale, *EORTC QLQ-C30* The European Organization for Research and Treatment of Cancer 30-item quality of life questionnaire, *QOL* quality of life, *K-FCRI* Korean version of fear of cancer recurrence index. *P values for trend were obtained by linear regression analysis after adjusting for age, sex, cancer site, marital status, cancer stage, treatment modality, type of care providers and duration since cancer diagnosis. ^a^Others included thyroid disease, liver disease, lung disease, osteoporosis, lymphedema, peripheral neuropathy, and anemia. ^b^Higher score means better health status. ^c^Lower score means better health status

Independent associations between levels care coordination level and clinical outcomes, HRQoL and FCR are presented in Table 4. In general, with decreasing level of care coordination, probability of worse clinical outcomes, HRQoL and FCR increased. Compared to the coordinated care group, the uncoordinated group showed significantly higher risk of new comorbidity development after cancer diagnosis, more frequent clinic visits (≥ twice per year), lower EQ-VAS, lower global and functional scale of EORTC QLQ-C30, greater severity of FCR, higher psychological distress and poor coping strategies. The intermediate group showed higher risk of having lower level of EQ-VAS, global health status/QOL, role function, emotional function in EORTC QLQ-C30, more severe FCR, higher psychological distress, and poorer coping strategies compared to the coordinated group. When we did sensitivity analysis to evaluate the association between outcome variables of continuous score with care coordination level using linear regression model, EQ-VAS, global and functional scale of EORTC QLQ-C30 except social function, and coping strategies and reassurance of FCR were positively associated with care coordination. K-FCRI components except functional impairment were inversely associated with care coordination (Additional file 1: Table S3).

**Table 4:** Association^a^ between the level of satisfaction to care coordination and worse clinical outcomes, poor quality of life and fear of recurrence

Clinical outcomes (cut-off level)	Coordinated group (reference)	Intermediate group	Uncoordinated group	*P*-trend^a^
	OR	OR (95% CI)	OR (95% CI)	
New comorbidity after cancer diagnosis (≥1)	1	1.16 (0.79,1.69)	1.73 (1.02,2.92)	0.013
Multiple clinic visiting (≥2)	1	1.18 (0.83,1.68)	1.69 (1.00,2.88)	0.010
Quality of life indices (cut-off level)				
Low EQ_VAS (< 60)	1	1.41 (1.10,1.82)	1.82 (1.28,2.60)	0.001
Low level of EORTC QLQ-C30				
Low Global health status/QOL (< 50.00)	1	1.45 (1.10,1.89)	1.51 (1.04,2.21)	0.012
Low physical functioning (< 73.33)	1	1.31 (0.96,1.80)	2.00 (1.31,3.04)	0.005
Low role functioning (< 66.67)	1	1.73 (1.31,2.27)	2.46 (1.69,3.56)	< 0.001
Low emotional functioning (< 66.67)	1	1.91 (1.46,2.48)	2.62 (1.81,3.78)	< 0.001
Low cognitive functioning (< 66.67)	1	0.99 (0.77,1.26)	1.57 (1.11,2.22)	0.025
Low social functioning (< 66.67)	1	1.30 (0.76,2.24)	2.07 (1.42,3.03)	0.001
K-FCRI				
Triggers (≥17)	1	1.46 (0.95,2.25)	1.90 (1.06,3.42)	0.062
Severity (≥16)	1	1.68 (1.14,2.49)	2.28 (1.33,3.93)	0.003
Psychological distress (≥7)	1	1.68 (1.06,2.66)	2.47 (1.35,4.52)	0.008
Functional impairment (≥8)	1	1.12 (0.72,1.74)	1.24 (0.67,2.28)	0.758
Coping strategies (< 15)	1	2.51 (1.52,4.14)	2.49 (1.27,4.88)	< 0.001
Insight (≥2)	1	0.69 (0.39,1.21)	0.74 (0.33,1.63)	0.410
Reassurance (< 2)	1	1.45 (0.83,2.54)	1.36 (0.61,3.00)	0.410

*OR (95%CI)* odds ratio (95% confidence interval), *EQ-VAS* EuroQoL Visual Analogue Scale, *EORTC QLQ-C30* The European Organization for Research and Treatment of Cancer 30-item quality of life questionnaire, *QOL* quality of life and *K-FCRI* Korean version of fear of cancer recurrence index

We defined the cut-off level of low EQ_VAS, low EORTC QLQ-C30 and increased fear of cancer recurrence as the forth quartile of them

^a^Evaluated by multiple logistic regression analysis after adjusting for age, sex, cancer site, marital status, cancer stage, treatment modality, type of care providers and duration since cancer diagnosis

## Discussion

In this study of adult Korean cancer survivors, we found that perception of care coordination varied according to clinical and socio-demographic characteristics of cancer survivors. Cancer survivors’ perception of care coordination was associated with diverse outcomes. In our study, cancer survivors having higher level of care coordination that was provided to them during cancer treatment were more likely to have favorable outcomes regarding comorbidity development, medical utilization, HRQoL and fear of cancer recurrence than uncoordinated survivors.

Well-coordinated care reflects a good relationship between patients and healthcare providers. A provision of adequate patient education on lifestyle modification has been found to be effective for preventing comorbidity development [20]. A systematic review of studies mainly conducted in the USA showed that well-coordinated cancer care was associated with more appropriate health care utilization (OR, 1.9; 95% CI: 1.5–3.5) compared to usual care [3]. Similarly, our study showed that survivors with care coordination for their cancer treatment tended to have decreased comorbidity development with fewer clinic visits than survivors with uncoordination, although the number of comorbidities prior to cancer diagnosis was not significantly different between the two groups. Such relation between levels with care coordination for health care utilization and health outcome could be explained by findings of our study showing that well-coordinated cancer care is closely associated with receipt of whole person care and tailored patient education for lifestyle modification. Well-coordinated care by the medical team with timely assessment and preventive intervention may reduce the development of new comorbidities [20]. These findings are consistent with previous results showing that care coordination can prevent long-term or late adverse effect after cancer diagnosis and planned treatment [21]. Although an association between coordination of cancer care and HRQoL or psychological distress was not evident in a study performed in the USA [3], the present study found that cancer survivors’ perception toward care coordination had a significant association with emotional function of HRQoL, psychological distress and severity of FCR. This finding suggests that more frequent clinic visits of the uncoordinated group might be associated with their lower HRQoL and higher FCR arising from psychological distress. Further study was needed to evaluate the association between perception toward care coordination and cut-off of FCR in Korean cancer survivors.

We measured participants’ care coordination using two questions. The first question was: “who played a care coordinator role for your cancer treatment?’ It was used to evaluate the established accountability or responsibility across care settings. It can measure the process of monitoring, follow-up and information transferring [12]. The second question was: “Did you receive all care services that were necessary for dealing with your health concern?” This was used to align resources with patient needs and support self-management goals [12]. Care coordination is a kind of psychological process and a complex measure influenced by perceived quality of care and individual expectations for provided care and outcomes of care [22, 23]. Thus, it could be a complicated matter to clarify characteristics of survivors who feel that they had received uncoordinated care. It might be also influenced by sociodemographic factors and clinical factors (including cancer type or duration since cancer diagnosis) as well as cultural differences [11]. In the present study, we found that, among nonclinical factors, demographic factors such as age, sex and marital status were closely related with the level of perceived care coordination. The cancer survivors who were younger, females, diagnosed with cancer within the last 5 years, and without a marital partner may be more likely to suffer from psychological distress of cancer patients [24]. However, socioeconomic status was not a significant factor associated with care coordination. The health care system of Korea where about 95% Koreans have a national health insurance may explain this finding. The Korea National Health Insurance System provides cancer patients with extensive (95%) compensation for treatment of cancer and cancer-related medical conditions. This markedly relieved health care expenses of cancer patients.

Health outcomes could be affected by several types of care coordination such as a designated role of care coordinator or navigator, survivorship care plan, described plans and regularly scheduled meeting times [6]. For example, telehealth system for improving communication between a care coordinator and patients can increase HRQoL of newly diagnosed cancer patients who are receiving chemotherapy [25, 26]. Intervention with utilization of nursing care for patients after abdominal surgery for ovarian cancer can reduce emergency department visits at urgency, although it does not affect hospitalizations or oncology outpatient visits [27]. In our study, perception of care coordination was found to vary according to the type of care providers, but the influence by the type of specialist involved in coordinated care on health outcomes of cancer survivors could not be assessed. To enhance effective flow of information between clinicians and patients in response to physical, psychological, social and spiritual needs of cancer survivors, several coordination strategies considering differences in culture and medical settings of patients’ population have been proposed, including face-to-face communication, electronic medical record, paper based and structured survivorship care plan [6, 28]. However, it remains uncertain which one is the best strategy to improve care coordination among those models and interventions [6, 7]. In the present study, we found that cancer survivors having uncoordinated care were less likely to perceive that they had received whole person care across all areas (including physical, psychological, social and spiritual care) compared to those in the coordinated group. In addition, those in the uncoordinated group perceived that they had less good communication within medical team compared to those in the coordinated group. To evaluate the influence of coordination strategies considering culture and medical settings, a larger study involving diverse population from different health systems and medical settings is warranted.

In Korea, given the current oncology practice pattern, an institution-based shared care model could be a potential solution to improve care coordination. The Korean National Cancer Control Institute has been developing ‘integrative supportive care service delivery system for cancer survivors’ since 2010 and a national pilot project for care coordination are currently ongoing between 11 regional cancer centers and public health centers since July, 2017 [29].

The present study has some limitations. First, participants in our study may not represent all cancer survivors at various settings because they were recruited from survivorship clinics of two academic hospitals. However, given that the two institutions are involved in cancer care of around 20% of all Korean cancer patients and cancer survivors of various types with various stages of cancer have been enrolled for this study, representativeness of our study might not be so serious. In addition, there was no difference in age and sex distribution, time lapse after cancer diagnosis, and cancer treatment modality between the participants of our study and the cancer survivors who refused to participate in our study (Additional file 1: Table S1). Second, the assessment of perception of care coordination has not been validated previously. Thus, findings of our study need to be cautiously interpreted in other setting. Third, time relation between cancer diagnosis and development of comorbidity could be inaccurate because these data were obtained cross-sectionally. We tried to overcome the reverse causation problem by checking newly developed comorbidity after cancer diagnosis and the current clinic visiting, but temporal relationship between perception of care coordination and health outcome could be still uncertain. A prospective approach to evaluate the relationship between care coordination and patient outcomes would be needed. Fourth, the outcome variables assessed in the current study are still only part of all clinical endpoints. We could not evaluate some important aspects of patient outcomes such as self-efficacy, knowledge, screening for the second primary cancer, or health promotion activities [22]. Fifth, we could not consider healthcare system factors because more than 95% Korean population have been provided with the National Health Insurance service since 1989 and their medical expenses for treatment of cancer and cancer-related medical conditions are extensively (95%) compensated. Lastly, we could not evaluate the effect of quality of clinic or provider because we could not recruit study subjects from a range of medical settings [30].

## Conclusion

The present study showed that Korean cancer survivors’ perception of care coordination was closely associated with care contents. These findings suggest that comprehensive care contents such as whole person care, good communication within medical team and tailored patient education may be reinforced to improve outcomes of survivorship care.

## Supplementary information

**Additional file 1: Table S1.** Comparisons of demographic and clinical characteristics between participants and non-participants. **Table S2.** Distribution of baseline and new comorbidities according to the level of care coordination. **Table S3.** Percent difference (95% confidence interval) of quality of life indices and fear of cancer recurrence associated with increase in level of care coordination.

## Data Availability

The datasets used and/or analyzed during the current study are available from the corresponding author on reasonable request.

## Abbreviations

EORTC QLQ-C30: European Organization for Research and Treatment of Cancer Quality of Life Questionnaire version 3.0
EQ-VAS: EuroQoL-visual analogue scale
FCR: Fear of cancer recurrence
HRQoL: Health-related quality of life
K-FCRI: Korean version of fear of Cancer Recurrence Index
