# Supplementary material for: Association between perception of care coordination and health outcomes in Korean cancer survivors
Source: Health Qual Life Outcomes. 2020 Feb 4;18:21. doi: 10.1186/s12955-020-1279-6 (PMC7001372; doi:10.1186/s12955-020-1279-6)
Supplement: Supplementary file 1 — Additional file 1: Table S1. Comparisons of demographic and clinical characteristics between participants and non-participants. Table S2. Distribution of baseline and new comorbidities according to the level of care coordination. Table S3. Percent difference (95% confidence interval) of quality of life indices and fear of cancer recurrence associated with increase in level of care coordination. [file 12955_2020_1279_MOESM1_ESM.docx]

| **Supplementary Table 1.** Comparisons of demographic and clinical characteristics between participants and non-participants | | | |
| --- | --- | --- | --- |
| Variables | Participants  (n=1,306) | Non-participants  (n=268) | P-value* |
| Mean age, years | 57.3±12.5 | 57.8±10.1 | 0.689 |
| Female | 62.2 | 67.2 | 0.164 |
| Mean year since cancer diagnosis | 5.80±1.9 | 5.84±2.6 | 0.835 |
| Cancer site |  |  | <0.001 |
| Stomach | 40.4 | 27.6 |  |
| Breast | 27.6 | 25.0 |  |
| Lung | 9.1 | 10.1 |  |
| Thyroid | 7.1 | 11.2 |  |
| Colon/rectum | 4.3 | 6.7 |  |
| Prostate | 2.5 | 1.5 |  |
| Others | 9.0 | 17.9 |  |
| Treatment modality |  |  |  |
| Surgery | 93.0 | 91.6 | 0.441 |
| Surgery & Chemotherapy | 34.4 | 38.2 | 0.206 |
| Surgery & Radiotherapy | 29.0 | 34.7 | 0.074 |

Data were presented as mean± standard deviation or percentage.

*assessed by t test or chi-square test.

| **Supplementary Table 2**. Distribution of baseline and new comorbidities according to the level of care coordination | | | | | | | | |
| --- | --- | --- | --- | --- | --- | --- | --- | --- |
| Comorbidity | Overall (N=1306) | | Uncoordinated  group  (N=167) | | Intermediate  group  (N=494) | | Coordinated  group (N=645) | |
|  | Baseline*  N (%) | New†  N (%) | BaselineN (%) | New  N (%) | BaselineN (%) | New  N (%) | BaselineN (%) | New  N (%) |
| Total | 232(17.8) | 396(30.3) | 25(15.0) | 55(32.9) | 103(20.9) | 159(32.2) | 104(16.1) | 182(28.2) |
| Cerebrovascular disease | 8(0.6) | 16(1.2) | 0 | 3(1.8) | 1(0.2) | 8(1.6) | 7(1.1) | 5(0.8) |
| Hypertension | 62(4.7) | 65(5.0) | 4(2.4) | 10(6.0) | 26(5.3) | 26(5.3) | 32(5.0) | 29(4.5) |
| Diabetes mellitus | 35(2.7) | 23(1.8) | 3(1.8) | 1(0.6) | 14(2.8) | 9(1.8) | 18(2.8) | 13(2.0) |
| Dyslipidemia | 29(2.2) | 105(8.0) | 6(3.6) | 15(9.0) | 12(2.4) | 39(7.9) | 11(1.7) | 51(7.9) |
| Mental disorder | 20(1.5) | 14(1.1) | 3(1.8) | 2(1.2) | 12(2.4) | 4(0.8) | 5(0.8) | 8(1.2) |
| Others‡ | 78(6.0) | 173(13.2) | 9(5.4) | 24(14.4) | 38(7.7) | 73(14.8) | 31(4.8) | 76(11.8) |

*Comorbidity at the time of cancer diagnosis. †Comorbidity newly developed after cancer diagnosis

‡ Others included thyroid disease, liver disease, lung disease, osteoporosis, lymphedema, peripheral neuropathy, and anemia

**Supplementary Table 3.** Percent difference (95% confidence interval) of quality of life indices and fear of cancer recurrence associated with increase in level of care coordination

| Clinical outcomes | % difference (95% CI)* | *P*-value* |
| --- | --- | --- |
| Quality of life indices | | |
| EQ_VAS | 5.4(2.5,8.4) | <0.001 |
| EORTC QLQ-C30 | | |
| Global health status/QOL | 5.5(2.2,9.0) | 0.001 |
| Physical functioning | 3.0(0.9,5.2) | 0.005 |
| Role functioning | 6.0(3.3,8.8) | <0.001 |
| Emotional functioning | 6.5(3.4,9.7) | <0.001 |
| Cognitive functioning | 2.9(0.1,5.8) | 0.043 |
| Social functioning | 1.6(-1.6,4.9) | 0.321 |
| K-FCRI | | |
| Triggers | -9.4(-14.5,-4.0) | 0.001 |
| Severity | -12.1(-17.5,-6.5) | <0.001 |
| Psychological distress | -13.3(-20.3,-5.8) | 0.001 |
| Functional impairment | -8.7(-17.3,0.80) | 0.072 |
| Coping strategies† | 11.2(5.5,17.0) | <0.001 |
| Insight | -11.8(-20.3,-2.2) | 0.017 |
| Reassurance† | 10.1(2.7,17.9) | 0.006 |
| 95 % CI: 95% confidence interval, EQ-VAS: EuroQoL Visual Analogue Scale, EORTC QLQ-C30: The European Organization for Research and Treatment of Cancer 30-item quality of life questionnaire, QOL: quality of life, K-FCRI: Korean version of fear of cancer recurrence index.  * β coefficients (95% confidence interval) for log-transformed HRQoL and K-FCRI associated with increase in level of coordinated care were estimated by linear regression model after adjusting for age, sex, cancer site, marital status, cancer stage, treatment modality, type of care providers, and duration since cancer diagnosis. Then, percent difference of HRQoL and K-FCRI was calculated by multiplying 100 to the value of exponentiated β coefficient -1. | | |
